# High recovery of cell-free methylated DNA based on a rapid bisulfite-treatment protocol

**DOI:** 10.1186/1471-2199-13-12

**Published:** 2012-03-26

**Authors:** Inge Søkilde Pedersen, Henrik Bygum Krarup, Ole Thorlacius-Ussing, Poul Henning Madsen

**Affiliations:** 1 Section of Molecular Diagnostics, Department of Clinical Biochemistry, Aalborg University Hospital, Denmark, DK; 2 Department of Surgical Gastroenterology, Aalborg University Hospital, Denmark, DK

## Abstract

**Background:**

Detection of cell-free methylated DNA in plasma is a promising tool for tumour diagnosis and monitoring. Due to the very low amounts of cell-free DNA in plasma, analytical sensitivity is of utmost importance. The vast majority of currently available methods for analysing DNA methylation are based on bisulfite-mediated deamination of cytosine. Cytosine is rapidly converted to uracil during bisulfite treatment, whereas 5-methylcytosine is only slowly converted. Hence, bisulfite treatment converts an epigenetic modification into a difference in sequence, amenable to analysis either by sequencing or PCR based methods. However, the recovery of bisulfite-converted DNA is very poor.

**Results:**

Here we introduce an alternative method for the crucial steps of bisulfite treatment with high recovery. The method is based on an accelerated deamination step and alkaline desulfonation in combination with magnetic silica purification of DNA, allowing preparation of deaminated DNA from patient samples in less than 2 hours.

**Conclusions:**

The method presented here allows low levels of DNA to be easily and reliably analysed, a prerequisite for the clinical usefulness of cell-free methylated DNA detection in plasma.

## Background

Bisulfite induced modification of nucleic acids was originally described in the 1970s [1-3] and since the emergence of PCR and sequencing based detection methods [4,5] bisulfite treatment has played a pivotal role in the analysis of DNA methylation. In its original form it is a time consuming and labour intensive procedure involving numerous experimental steps: DNA denaturation, treatment with bisulfite for 12-16 hr, desalting and desulfonation with NaOH, and finally neutralisation and desalting. Recently published improvements include an accelerated deamination step, cutting down incubation time from 12-16 hr to 40 min, achieved by the use of a more concentrated bisulfite solution at higher temperatures [6,7]. The accelerated and the conventional methods have been explicitly compared by Genereux et al. [8]. The accelerated method leads to a more homogenous conversion of cytosine both across sites and molecules, conceivably due to facilitation of DNA denaturation in concentrated bisulfite solution at 70°C. Hence, inappropriate conversion of 5-methylcytosine, a result of prolonged bisulfite exposure of molecules with complete conversion of cytosine, is more controllable.

In addition to deamination of cytosine, bisulfite also induces chain breakage of DNA [9]. The DNA degradation caused by bisulfite treatment results in DNA fragments of an average length of approximately 600 nucleotides [10]. Real-time PCR based methods rely on amplification of short DNA fragments of 60-150 nucleotides. Hence, the use of real-time PCR limits the direct influence of fragmentation on the detection step. However, fragmentation affects recovery of DNA after bisulfite treatment. If the starting material is < 200 ng DNA, more than 95% of bisulfite-treated DNA is lost during desulfonation and purification with standard procedures [10,11]. This is a serious problem, especially when analysing material with very small amounts of DNA available, such as plasma with a median level of 10 ng cell-free DNA/ml in normal controls [11]. Although, the level of cell-free DNA is slightly increased in cancer patients [12], the combination of minute amounts of cell-free DNA in plasma and poor recovery after bisulfite treatment may lead to stochastic sampling issues. Improved recovery can be achieved by incorporation of DNA into agarose prior to bisulfite treatment. Denaturation, deamination and desulfonation are subsequently carried out on DNA embedded in the agarose beads [13]. This method has successfully been used to analyse DNA from microdissected cells [14]. Embedding of DNA in agarose is, however, a labour intensive method not amneable to automation, limiting its suitability in a clinical setting.

The ability to easily analyse sparse amounts of methylated DNA is a prerequisite for the usefulness of cell-free methylated DNA in plasma as a diagnostic or prognostic marker for cancer. An enormous amount of work has been put into identification of putative methylated DNA biomarkers and optimisation of the final analytical step: the detection of bisulfite-treated DNA. Hence, several reliable methods enabling the detection of minute amounts of bisulfite-treated DNA have been published [15-20]. Most recently the qMAMBA protocol (quantitative Methylation Analysis of Minute DNA amounts after whole Bisulfitome Amplification) has been developed, elegantly addressing several of the issues related to methylation analysis of samples with very low amounts of starting material [19,20]. However, as stated by Paliwal *et al. *[19] the most critical determinant of successful application of qMAMBA is the quality and quantity of starting material, emphasising the importance of the initial steps of the analysis: DNA isolation and bisulfite-treatment. Very few studies have addressed the loss of analytical sensitivity associated with the bisulfite treatment itself. Here we present a fast and reliable method, optimised in order to achieve high recovery, for the detection of methylated DNA from biospecimens with sparse amounts of DNA.

## Methods

### Bisulfite treatment

Cell-free DNA was isolated from 1 ml EDTA plasma on the EasyMAG nucleic acid purification platform (Biomeriéux), using the recommended protocol for plasma.

Bisulfite treatment was based on previously published accelerated methods [6,7], with some modifications. The optimised protocol is as follows: 50 μl of 10 M (NH_4_)HSO_3_-NaHSO_3 _solution were added to 25 μl of purified DNA in PCR strips. The strips were placed in a PCR machine, heated for 10 min at 90°C and subsequently cooled to room temperature. The solution containing the DNA-bisulfite adducts were purified on the EasyMAG nucleic acid purification platform (Biomeriéux) using the off-board procedure according to manufacture's instructions, except for changes made to lysis buffer, extraction buffers A and B, and elution buffer. To ensure high recovery, 2 ml EasyMAG lysis buffer were replaced by a mix of 0.5 ml ethanol and 0.5 ml H_2_O, whereas extraction buffers A and B (Biomeriéux) both were replaced by 33% ethanol in H_2_O. For this extraction 25 μl magnetic beads were used. Finally, DNA was eluted in 25 μl 10 mM KOH. The alkaline solution in combination with the heating occurring during elution leads to desulfonation of DNA-bisulfite adducts.

### DNA quantitation

**Figure 1:**
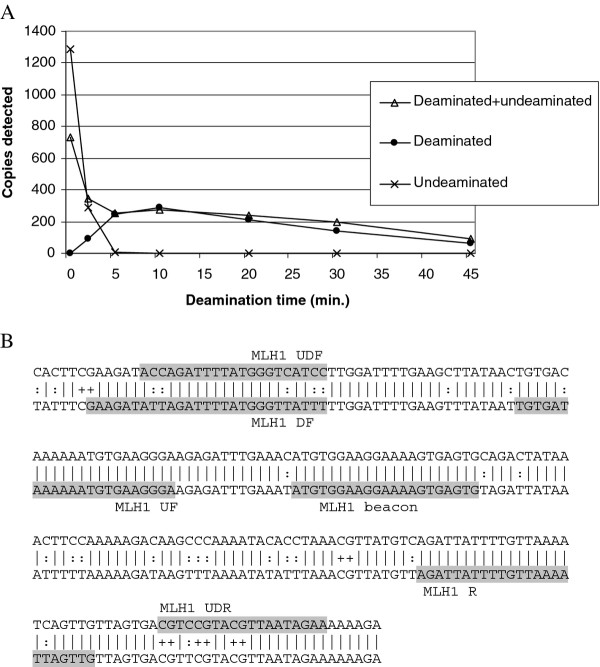
**Monitoring of reaction dynamics by real-time PCR**. A) Monitoring of the deamination reaction as a function of deamination time using primers designed for an unmethylated part of the *MLH1 *promoter. Three different primer sets were used. The first set (MLH1 UF and MLH1 R) binds to areas with no cytosines, and hence amplify both deaminated and undeaminated DNA. However, since deamination creates 2 uncomplementary DNA strands, only one strand of the deaminated DNA can function as a template. The second set (MLH1 DF and MLH1 R) cytosine has been replaced by thymine making it specific to deaminated DNA. The third set (MLH1 UDF and MLH1 UDR) contains cytosines making it specific to undeaminated DNA. Real-time PCR detection of the 3 products was performed with a common molecular beacon (MLH1 beacon). B) Binding sites of beacon and primers used are shaded. Top strand is the undeaminated DNA sequencing. Bottom strand is the deaminated sequence. A horizontal line between the two strands illustrates no difference in sequence, ":" marks the positions of cytosine converted to uracil during deamination, and "+" marks the positions of CpG dinucleotides. Primer sequences can be found in **Table **1. The universal primers, MLH1 UF and MLH1 R, contain Inosine at one position each.

To investigate recovery of bisulfite-treated DNA, 3 different primersets, all sharing the same detection probe, were designed: MLH1UF and MLH1 R detect DNA regardless of deamination [11]. MLH1 DF and MLH1 R detect deaminated DNA, whereas MLH1 UDF and MLH1 UDR detect undeaminated DNA (Table 1 and Figure 1B).

**Table 1:** Primer and beacon sequences

Primer/beacon	Sequence
MLH1 UF	TGT GAI AAA AAA TGT GAA GGG

MLH1 DF	GAA GAT ATT AGA TTT TAT GGG TTA TTT

MLH1 R	CAA CTI AAT TTT AAC AAA ATA ATC T

MLH1 UDF	ACC AGA TTT TAT GGG TCA TCC

MLH1 UDR	TTC TAT TAA CGT ACG GAC G

MLH1 beacon	(FAM)CGC GAA TGT GGA AGG AAA AGT GAG TGT CGC(Dabcyl)

RASSF1A MF	GGG AGG CGT TGA AGT C

RASSF1A MR	CCC GTA CTT CGC TAA CTT TAA ACG

RASSF1A M beacon	(HEX)CGC GAT TCG + TT + C G + GT TCG CTC GCG(Dabcyl)

RASSF1A UMF	TTT TGT ATT TAG GTT TTT ATT GTG T

RASSF1A UMR	CCC ATA CTT CAC TAA CTT TAA ACA

RASSF1A UM beacon	(FAM)CGC GAG + TT + TG + GTT + TG + TG + TTTC GCG(Dabcyl)

MEST MF	TGT CGC GGT AAT TAG TAT ATT TC

MEST MR	AAC CCG CGC AAA ACG ACG

MEST M beacon	(HEX)CGC GAT TAC + GAA AC + G CAA CTA CCG ATC GCG(Dabcyl)

MEST UMF	GTG TTG TTG TGG TAA TTA GTA TAT TTT

MEST UMR	AAC CCA CAC AAA ACA ACA CCA

MEST UM beacon	(FAM)CGC GAG + TA + G T + TG + TG + T TT + T GTT CGC G(Dabcyl)

MLH1 UF and MLH1 R concurrently amplify deaminated and undeaminated *MLH1 *promoter. MLH1 DF and MLH1 R amplify deaminated *MLH1*. MLH1 UDF and MLH1 UDR amplify undeaminated *MLH1 *promoter. A common molecular beacon (MLH1 beacon) was used for detection of the 3 products. RASSF1A MF and RASSF1A MR amplify methylated *RASSF1A*. RASSF1A UMF and RASSF1A UMR amplify unmethylated *RASSF1A*. Methylated and unmethylated products were detected by RASSF1A M beacon and RASSF1A UM beacon, respectively. MEST MF and MEST MR amplify methylated *MEST*, whereas MEST UMF and MEST UMR amplify unmethylated *MEST*. Methylated and unmethylated products were detected by MEST M beacon and MEST UM beacon, respectively. To ensure high specificity the probes designed to discriminate between methylated and unmethylated products contain LNA nucleotides at positions marked with +.

### Dynamics of deamination

Three μg of colon tissue DNA were deaminated in triplicate for different time durations, purified and desulfonated. Five ul DNA were used for quantitation, the residual DNA was used for HPLC. This DNA was digested with 1 unit nuclease P1 (Sigma) and 10 units DNAseI (Roche) in a total volume of 40 μl at 37°C overnight [21]. Two units alkaline phosphatase (Roche) were added and the mix was incubated at 37°C for 2 hours. Before injection, 50 μl of 50 mM sodium acetate pH 4.8 were added. Twentyfive μl were injected into a cation exchange column (Luna SCX, Phenomenex) in a Dionex Ultimate 3000 HPLC system. The mobile phase consisted of 5% acetonitrile in a gradient of 50 mM sodium acetate from 2% to 75%. Deoxynucleosides were detected at 260 nm. For calculation of relative amounts of nucleosides the following extinction coefficients have been used: dU (9.2*10^3 ^M^-1 ^cm^-1^), dT (9.2*10^3 ^M^-1 ^cm^-1^), dG (11.7*10^3 ^M^-1 ^cm^-1^), dA (15.4*10^3 ^M^-1 ^cm^-1^), dC (7.5*10^3 ^M^-1 ^cm^-1^) and dmC (7.5*10^3 ^M^-1 ^cm^-1^).

### Measurement of analytical sensitivity

The sensitivity of methylation detection was assessed by analysing a dilution series of Universal Methylated DNA (Chemicon) in purified plasma DNA. After deamination the recovered DNA was quantified in triplicate for methylated *RASFF1A *and unmethylated *RASSF1A *using primers and probes listed in Table 1.

The efficiency of the deamination of unmethylated cytosines and the recovery of both methylated and unmethylated DNA fragments following bisulfite-treatment were elucidated using the hemimethylated *MEST *promoter as a model. Plasma DNA was diluted to concentrations of 10, 5, 2.5 and 1.25 genome equivalents pr. 25 μl of methylated and unmethylated *MEST*, respectively. Twentyfour samples of each dilution were deaminated and used in methylation-specific real-time PCR with MEST UM primers and probe and MEST M primers and probe, respectively. Primer and probe sequences are listed in Table 1. The detection frequencies were calculated as the percentage of positive reactions out of the 24 replicates.

### Measurement of recovery

Recovery of deaminated plasma DNA was investigated using DNA from 24 plasma samples. DNA from 1 ml of each sample was extracted and eluted in 75 ul. Twentyfive ul were deaminated and extracted, another 25 ul were mock-deaminated (no heating) and extracted, whereas the residual was used for quantitation using *MLH1 *primers described in Table 1.

## Results and discussion

Extensive optimisation of a previously published bisulfite treatment method [6,7] has led to the development of a fast bisulfite treatment with high recovery and potential for semi-automation. Optimisation involved the purification procedure after deamination in order to facilitate recovery of the fragmented DNA resulting from bisulfite treatment. This part of the procedure has previously been identified as a critical step [10]. Optimisation of the purification method had a pronounced effect on recovery, especially replacement of lysis and extraction buffers with ethanol increased recovery. In addition, adjustments of the reaction time were made. During optimisation the dynamics of the reaction had to be measured in order to ensure optimal conversion of cytosine and limited conversion of methyl-cytosine.

**Figure 2:**
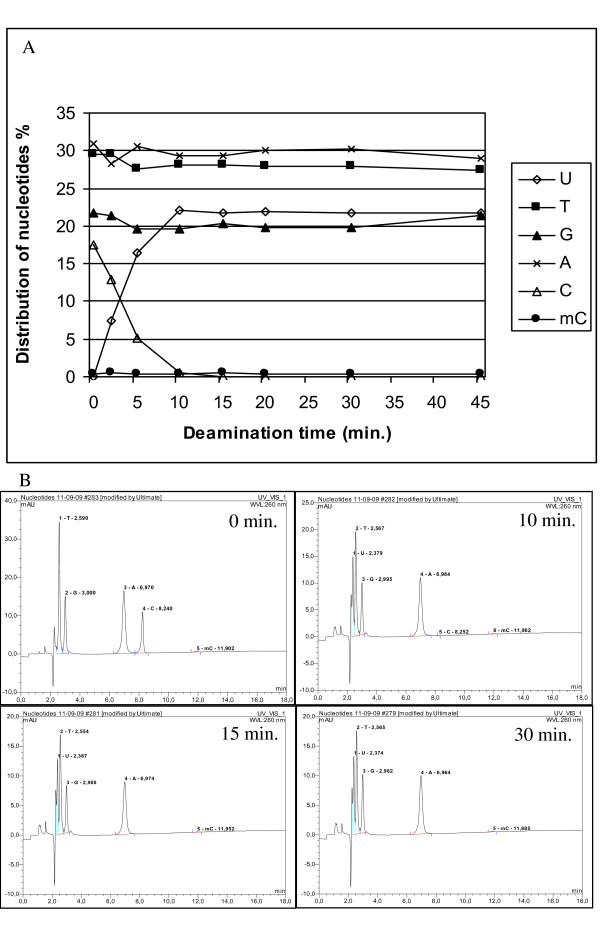
**Monitoring of reaction dynamics by HPLC**. A) The distribution of nucleosides as measured by HPLC depicted as a function of deamination time. The levels of Gs, As and methylated Cs remain constant throughout the reaction. Cs are converted to Us as the deamination progresses. B) Examples of HPLC chromatograms at different deamination times (0 min., 10 min., 15 min. and 30 min.).

In the discovery phase bisulfite treatment, cloning and sequencing of individual clones is the method of choice, because of the detailed information of methylation status of individual CpG sites achieved. This procedure, from primer design to presentation of results has been described in detail [22]. When sequencing individual clones, complete deamination of unmethylated cytosine is extremely important. Incomplete deamination of unmethylated cytosine could lead to a false positive result. Typically more than one methylated CpG site is investigated when using methylated DNA as a tumour marker. Hence, this set up is less likely to yield a false positive result due to incomplete conversion of cytosine. However, if the reaction is poorly optimised and conversion of cytosine is far from complete, real-time detection will fail, leading to apparently low recovery. Therefore, while optimising recovery, dynamics of the reaction have been extensively monitored using both a real-time PCR based and a HPLC based method. The results of optimisation of the reaction time are shown in Figures 1 and 2. After 5 min of deamination, no undeaminated product was detectable by real-time PCR (Figure 1). The beacon and primers used in this experiment have been designed in order to give as comparable reaction conditions as possible. The overlap in the probe and primers used for the different reactions gives less stringent differentiation compared with methylation specific real-time PCR, where both primers and probes generally are specific for either the methylated or the unmethylated sequence. However, no cross-reactivity is observed in our experiment. The primers used to discriminate between undeaminated and deaminated product cover 5 cytosines (Figure 1). Primer binding mainly depends on the conversion of the 2 cytosines in the 3' end of the primer binding site. Therefore complete conversion of cytosine appears to be achieved faster when measured by real-time PCR compared with HPLC, where a limited amount of cytosines (< 1%) is detectable after 10 min deamination, and none after 15 min (Figure 2). Longer deamination time than 10 min results in reduced detection of both deaminated and undeaminated DNA, since the purpose is to optimise this protocol in order to achieve the best possible recovery of bisulfite treated DNA, the deamination time was set to 10 min.

**Figure 3:**
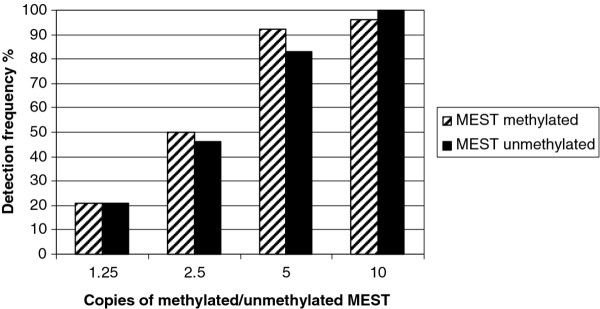
**Detection of the hemi-methylated *MEST *promoter**. Recovery of methylated and unmethylated *MEST*, measured in a dilution series of cell-free DNA from plasma. Each reaction was run 24 times, and the detection frequency is calculated as the percentage positive reactions.

In addition to incomplete conversion of cytosine, inappropriate conversion of methylated cytosine could lead to reduced sensitivity when detecting methylated DNA. The real-time PCR based experiment (Figure 1) does not address the problem of over-conversion, since the primers and the probe are designed to an unmethylated part of *MLH1*. Methylated cytosine is monitored by the HPLC procedure (Figure 2). However, methylated cytosine constitutes only a small fraction of the nucleotides. Therefore, to further elucidate the reaction dynamics and recovery of both methylated and unmethylated DNA, the hemimethylated *MEST *promoter has been used as a naturally occurring model system. Comparable detection of methylated and un-methylated *MEST *was observed, when calculating detection frequency as the percentage of positive reactions out of the 24 replicates (Figure 3).

Similar experiments monitoring the dynamics of the reaction were carried out when changing other parameters, such as reaction temperature and denaturation of DNA prior to bisulfite treatment (data not shown). Denaturation of DNA did not affect recovery or conversion in the current study, even though this step has been pointed out to be critical, and different denaturants such as NaOH, urea and formamide have been used. Formamide was shown to have a great effect when working on DNA from formalin-fixed paraffin-embedded tissue [23]. The lack of need of denaturants in the protocol presented here possibly reflects the denaturating effect of the high concentration of bisulfite and high temperature used for deamination.

Further investigation of the analytical sensitivity of the newly optimised procedure was carried out using a dilution of universally methylated DNA in purified plasma DNA. *RASSF1A *was used as a model system. *RASSF1A *is fully methylated in the universally methylated DNA and unmethylated in the plasma DNA sample. Table 2 illustrates that low copy numbers of methylated *RASSF1A *promotor region was reliably detected in a background of approximately 2000 copies of unmethylated DNA.

**Table 2:** Detection of methylated DNA diluted in unmethylated DNA

Copies of methylated DNA	0	1.3	4	11.9	35.6	107	320	960
Detected methylated copies (*RASSF1A*)	0	0.5	4.9	11	41	142	328	832

Detected unmethylated copies (*RASSF1A*)	1748	2192	2297	2207	1832	2700	2285	2194

Detection of *RASSF1A *in a 3-fold dilution series of methylated DNA in a background of 2000 copies of DNA. Top row represents the calculated number of methylated copies of *RASSF1A *promoter. The following rows represent methylated and unmethylated copies respectively, detected by real-time PCR.

Recovery of deaminated DNA using the optimised method was compared with recovery of DNA in a matrix of deamination reagents. Re-extraction of DNA in a matrix of deamination reagents results in a recovery of 83% whereas recovery of deaminated DNA is 60% (Table 3). The likely explanation of the difference is the unavoidable degradation of DNA during deamination.

**Table 3:** Recovery of plasma DNA

	Average copy no. (copies/ml plasma)	Median copy no. (copies/ml plasma)	Recovery (%)^1^
Plasma DNA, double stranded	3629	1475	

Mock-deaminated DNA, double stranded	2830	1223	83.3

Deaminated DNA, single stranded	2437	667	60.5

^1^Recovery has been calculated as the average recovery of 24 individual deamination reactions.

Markers such as septin 9 hold great promise of clinical usefulness. The improved recovery rate of the bisulfite reaction achieved by the presented protocol may further improve its potential in a clinical setting, and avoid some of the expensive and labour intensive steps such as the need to purify multiple plasma samples in parallel and subsequent pooling of DNA [16,24]. Indeed, the initial promising reports on the potential of septin 9 have been strengthened by a recent report employing silica based magnetic DNA purification [25]. This work by deVos *et al. *address the problem of poor recovery, and the reported recovery rates are comparable to our results. However, the quantitation of DNA prior to and after bisulfite treatment is based on two different real-time PCR assays. We have employed a real-time PCR assay capable of detecting both treated and untreated DNA [11] allowing the same assay to be used for measuring DNA levels both pre and post bisulfite treatment resulting in improved accuracy

## Conclusions

In this report we present a fast and reliable bisulfite treatment with high recovery from samples with minute amounts of DNA (< 0.1 ng/ml), opening the possibility of improving diagnostic sensitivity of the wide-range of potential markers already identified. The major improvement in recovery is achieved by alterations in the purification of bisulfite treated DNA. In addition, the method presented here allows preparation of deaminated DNA from patient samples in less than 2 hours. The development of this method amenable to semi-automation should considerably enhance the usefulness of methylation analysis in a clinical setting.

## Competing interests

The authors declare that they have no competing interests.

## Authors' contributions

ISP participated in study design and analysis and drafted the manuscript. HBK and OTU participated in study design and helped to draft the manuscript. PHM participated in study design, carried out the analyses and helped to draft the manuscript. All authors read and approved the final manuscript.
